# Early adversity predicts adoptees’ enduring emotional and behavioral problems in childhood

**DOI:** 10.1007/s00787-020-01553-0

**Published:** 2020-05-28

**Authors:** Amy L. Paine, Kevin Fahey, Rebecca E. Anthony, Katherine H. Shelton

**Affiliations:** 1 School of Psychology, Cardiff University, Tower Building, 70 Park Place, Cardiff, CF10 3AT UK; 2 Department of Political and Cultural Studies, Swansea University, James Callaghan Building, Sketty, Swansea, SA2 8PZ UK; 3 Centre for the Development and Evaluation of Complex Interventions for Public Health Improvement (DECIPHer), School of Social Sciences, Cardiff University, 1-3 Museum Place, Cardiff, CF10 3BD UK

**Keywords:** Adoption, Risk, Mental health, Childhood, Longitudinal study

## Abstract

Children adopted from the public care system are likely to experience a cluster of inter-related risk factors that may place them on a trajectory of mental health problems that persist across the life course. However, the specific effects of putative risk factors on children’s mental health post-placement are not well understood. We conducted a prospective, longitudinal study of children placed for adoption between 2014 and 2015 (*N* = 96). Adoptive parents completed questionnaires at approximately 5, 21, 36, and 48 months post-placement. We used time series analysis to examine the impact of pre-adoptive risk factors [adverse childhood experiences [ACEs], number of moves, days with birth parents and in care] on children’s internalizing and externalizing problems, and prosocial behavior over 4 years post-placement. Adoptees’ internalizing and externalizing problems remained consistently high over the 4-year study period, but more ACEs predicted increases in internalizing and externalizing problems. Contrary to expectations, more pre-placement moves and time in care predicted fewer problems over time, but exploratory analyses of interactive effects revealed this was only the case in rare circumstances. We identify pre- and post-removal factors that may incur benefits or have a deleterious impact on adoptees’ outcomes in post-adoptive family life. Our findings provide knowledge for front-line professionals in the support of adoptive families and underscore the vital need for effective early intervention.

**Electronic supplementary material:**

The online version of this article (10.1007/s00787-020-01553-0) contains supplementary material, which is available to authorized users.

## Introduction

An accumulation of early risk factors heralds numerous negative developmental outcomes including emotional (internalizing; anxious, withdrawn, and depressed) and behavioral (externalizing; disinhibition and aggression) problems [1]. For adopted children, early risk factors may include birth family history (e.g. birth mother and/or father medical and/or psychological problems), prenatal risk factors (e.g. maternal drug/alcohol misuse, stress, poor prenatal care, birth complications) and/or postnatal adversity (e.g. abuse, neglect, instability) which may occur at crucial stages in development [2, 3] and place them at a higher risk for enduring developmental problems [4]. Studies investigating the mental health of, primarily, US adoptees, demonstrate that adopted children are at a greater risk for emotional and behavioral problems than non-adopted children, and that there is an overrepresentation of adoptees in mental health settings [5–7].

Most adopted children in the UK are taken into care due to maltreatment within the birth family [8], and are likely to have experienced adverse childhood experiences (ACEs) such as abuse, neglect, and household dysfunction than the general population [3, 9]. Recent work revealed that over two-fifths of children adopted in a 1-year period experienced four or more ACEs [9], with such exposure linked to an increase in vulnerability for health risks across the life course, such as delinquency in adolescence [10] and poor psychological health, alcoholism, drug abuse, and suicide attempts in adulthood [11–13].

In the UK, most children spend time in temporary kinship care or with foster carers prior to adoption [14]. Although this arrangement ceases the immediate risk of harm to a child, during this transition all adopted children experience the loss of their birth parents, and potentially extended family, friends, possessions, home, and community. Early adversity and loss may be also compounded by placement instability following the child’s removal. Repeated separations and unstable and unpredictable living arrangements also impact a child’s well-being [15]; evidence suggests placement instability influences a child’s behavioral problems irrespective of their pre-existing attributes and problems following their removal [16].

Adopted children’s pre-placement adversity is often estimated using age at adoption as a proxy of pre-placement risk. Children who are older at the time of adoption are more likely to experience psychological and behavioral problems, with children placed in their adoptive homes over the age of 4 being the most troubled [17, 18]. Older children placed for adoption are likely to have entered care at an older age, to have accumulated more pre-placement risk factors, including ACEs, and are at greater risk for their adoptive placement breaking down, termed a disruption [9, 19]. Although age at placement has been used to predict children's outcomes in recent work [20], age at adoption as a sole indicator of pre-adoptive adversity is problematic. Not only are associations with children’s outcomes inconsistent [21, 22], but implicit in the use of age at adoption is the assumption of a linear relationship between time before placement and the magnitude of pre-placement adversity [22]. As such, the influence of age at adoptive placement on children’s psychological and behavioral outcomes must be examined in combination with other pre-placement risk factors [6].

## The present study

We examined the impact of pre-adoptive risk factors on adopted children’s mental health problems over 4 years following adoptive placement. We extend prior work in several ways: we tested the relative contribution of multiple inter-related putative risk factors, including cumulative ACEs, number of moves, and number of days with birth parents and days in care to children’s internalizing and externalizing problems. Although children’s withdrawn and disruptive behavior is likely of most concern to parents and professionals, the absence of prosocial behavior can lead to peer rejection and associated negative sequelae [23]. Therefore, we also extended prior work by investigating relationships between risk factors and children’s prosocial behavior. We used time series analysis to overcome the pitfalls of data collection over multiple time points (e.g. data points taken over time may have an internal structure, such as autocorrelation). We hypothesized that ACEs and number of moves, over and above other pre-placement risk factors, would be associated with a greater risk for enduring internalizing and externalizing problems over 4 years post-placement. We controlled for child-related factors, such as gender, socioeconomic factors, and adoptive family structure.

## Method

### Design

The Wales Adoption Cohort Study (WACS) used a prospective, longitudinal mixed-methods approach to understand the early support needs and experiences of 96 newly formed adoptive families. Local authority adoption teams across Wales  were asked to send out letters on behalf of the research team to every family with whom they had placed a child for adoption from 01 July 2014 to 31 July 2015. The 96 families who returned the initial questionnaire at 5 months post-placement were followed up longitudinally over four time points post-placement. The present study focuses on the questionnaire follow-ups that took place at approximately 5, 21, 36, and 48 months post-placement [Waves 1–4 (W1–4), respectively]. Of the 96 families who participated in the study at W1, 81 (84.4%) participated at W2, 73 (76.0%) participated at W3, and 68 (70.8%) participated at W4. This study was conducted in accordance with the 1964 Declaration of Helsinki and its later amendments. Ethical permission for the study was granted by the Research Ethics Committee for the School of Social Sciences at Cardiff University and permission to access social work records was obtained from the Welsh Government (see [9] for more details).

### Background of adoption in the United Kingdom

Currently in the United Kingdom, the Children Act 1989 (UK) and the Social Services and Well-being (Wales) Act 2014 (Welsh Assembly) provide the legal framework for a child being supported within his or her family and community, establishing the local authority’s duties and court powers. The Adoption and Children Act 2002 (UK), with some minor amendments, sets out the legal framework for adoption in Wales. Most children will have been removed from their birth family into care if they are deemed to be at significant risk of harm and a care order is put forward that indicates adoption is the only appropriate option for the child’s needs. If the court endorses the care plan, a placement order is made that remains through matching and introductions to prospective adoptive parents, until they are authorized to move to their adoptive placement. The prospective adopters can apply for an adoption order 10 weeks following the child’s move to the placement. Once the adoption order is made, full parental responsibility is granted to the adoptive parents [14].

### Procedure

#### Social worker records

Within Wales, every local authority is mandated to complete a child adoption report (CAR), for each child where there is a plan for adoption, as set out in the Adoption Act Regulations (2005). CARs are completed by social workers, who record information based on their work with birth parents, contact with foster carers, liaison with other professionals (e.g. police, health visitors, and medical officers), and reviews of historical social services records. Baseline data concerning the characteristics and pre-adoptive history of each child were obtained by reviewing these records. Researchers worked on-site at the local authority offices and gathered information pertaining to the pre-adoptive history of the child and the age at which the child was moved into their permanent placement from electronic and hard-copy formats of CARs.

#### Questionnaires

At each time point, families completed a questionnaire concerning socio-demographic information, pre- and post-adoption experiences, the child and adoptive parent’s mental health, and adoptive family relationships. Where groups of siblings were placed together, parents were asked to report on the eldest child in the placement. Questionnaires were completed by either an adoptive mother (87.5% at W1, 87.7% at W2, 97.3% at W3, 92.6% at W4) or father. It was encouraged that the questionnaires should be completed by the same parent at each wave, so all families who provided follow-up questionnaires returned at least one completed by the same informant.

### Participants

Of the children who were reported on by their parents in the longitudinal follow-up questionnaires (*n* = 96), 47 (49%) were female and were placed for adoption at a mean age of 2.36 years (SD 2.20, range 0–9 years); 41.2% were removed at birth. Children spent a mean of 522.92 (SD 611.74, range 0–2344) days with their birth parents and a mean of 537.09 (SD 285.74, range 203–1401) days in care. Children experienced a median of 1 move (range 0–13). Twenty-nine children (30%) were adopted as part of a sibling group.

The adoptive parents in the study were a mean age of 40.67 (SD 6.98, range 22–62) years at the time of adoption, and the majority (99%, *n* = 94) were white British. Most parents were in a heterosexual relationship (82%, *n* = 79), 5% (*n* = 5) were in a same sex relationship and 13% (*n* = 12) were single adopters. At the W1 assessment, there was a median of 4 (range 2–7) people living in the household and most informants were in either full-time or part-time paid work (*n* = 72, 54.2%). Gross family income and education levels were substantially higher than the UK average (see [9]); 12% earned more than £75,000 per year and 37% had postgraduate degrees.

#### Sample representativeness

Characteristics of the 96 adopted children in the present study were compared to those of all children placed for adoption in Wales in the same time window (*N* = 374), by reviewing CARs for all children adopted between July 2014 and July 2015 in Wales. The sample was representative of children placed for adoption in this 13-month period regarding gender and past experiences of abuse and neglect (*p*s > 0.05). However, it contained slightly older children, because we asked parents of sibling groups (30% of the sample) to comment on the oldest child they had adopted. Attrition analyses showed no differences in socio-demographic characteristics (child gender and age, parent relationship status, education, and income) between those who participated in W1 and 4 of the study (all *p*s > 0.05).

### Measures

#### Pre-adoptive risk factors

Information regarding child characteristics (gender and date of birth) and their pre-adoptive background were obtained from review of each child’s CAR. Pre-adoptive risk factors included: (1) child’s age at placement in years; (2) number of days spent with birth parents; (3) number of days in care; (4) number of moves, defined as any change in placement recorded by the child’s social worker prior to their adoptive placement; and (5) number of adverse life experiences (ACEs) out of ten categories, see [9, 11], including childhood abuse (emotional, physical, or sexual), neglect, and household dysfunction (domestic violence, parental separation, substance abuse, alcohol abuse, mental illness, or incarceration). Each category was coded as absent (0) or present (1) resulting in an ACEs score for each child out of 10 ACEs.

#### Child internalizing and externalizing problems

Adoptive parents completed the Strengths and Difficulties Questionnaire [24]. We used internalizing (sum of emotional and peer problem scales), externalizing behavior problems (sum of conduct and hyperactivity scales), and prosocial behavior as our key outcome variables. A higher score is indicative of more problems for all subscales, except for the prosocial scale, where higher scores correspond to strengths in prosocial behavior (where children could score a maximum of 20 for internalizing and externalizing, and 10 for prosocial). The internalizing, externalizing, and prosocial scales had acceptable to good levels of internal consistency across all time points (*α*s ranged from 0.60 to 0.84).

#### Adoptive parent socioeconomic status and family structure

Adoptive parents’ socio-demographic information was collected at W1. Variables included: (1) adoptive parent (questionnaire informant) age at time of adoption; (2) adoptive parent relationship status (1 = single adopter, 2 = couple adopter); (3) adoptive parent highest level of education attained (1 = postgraduate or higher degree, 0 = other); (4) adoptive parent employment status (1 = full-time or part-time paid, 0 if other); and (5) gross family income (1 = up to £19,999, 2 = £20,000 to £49,999, 3 = £50,000 +). We also coded whether children were adopted alone into the household or whether other children were in the household (1 = any sibling, 0 = no sibling).

### Statistical analysis

#### Modelling time

The combined dataset consisted of 318 observations across four waves with a mean inter-wave attrition rate of 10.78 percentage points. The unit of observation was the respondent at each wave. We reported estimated coefficients for internalizing and externalizing problems, and prosocial behavior separately. Our estimation technique was ordinary least squares regression, accounting for serial correlation by employing time series analysis [25, 26]. Serial correlation violates the assumption that observations are independent; errors associated with an individual at time_*t*_ are positively correlated with errors at time_*t*−1_. We employed three estimation techniques to overcome the problems with serial correlation, specifically: (1) time period and unit-fixed effects; (2) the autoregressive distributive partial-adjustment lag model (AR-1); (3) an AR-1 model that only uses respondents who participated in all four waves (4-wave AR-1). Fixed-effects models account for unobserved heterogeneity across time and individual by providing each time period and individual with its own unique intercept. We can then estimate the effect of our predictors of interest without concerns about time or individual trends. By contrast, AR-1 models control for time by including the individual's outcome—from the previous time-period—as a predictor. Doing so controls for the different "starting points" for each individual child and permitted us to estimate the outcome without concerns pertaining to each individual having a different "starting point." Therefore, we estimated coefficients of predictors on the outcome controlling for the development of the child as a function of time.

This analysis permitted comparison between long-run effects (the pre-adoptive experiences of adopted children in the study) and short-run effects, (changes in the employment status of respondents or the unobserved contemporaneous well-being of the child). We were unable to conclusively test for co-integration due to sample size limitations and the relatively small number of waves in the study; therefore, we did not use error-correction models. Moreover, because many meaningful covariates were time invariant, we did not advocate for or use a differenced model.

#### Multiple imputation

We used the Amelia II R package to impute missing data [27]. We restricted imputed data to positive integers or zero. We also imputed our outcome variables when the respondent completed the questionnaire, but did not fill out all questions related to our constructed outcome variables; see the online supplement for robustness checks that do not impute missing data.

## Results

**Fig. 1:**
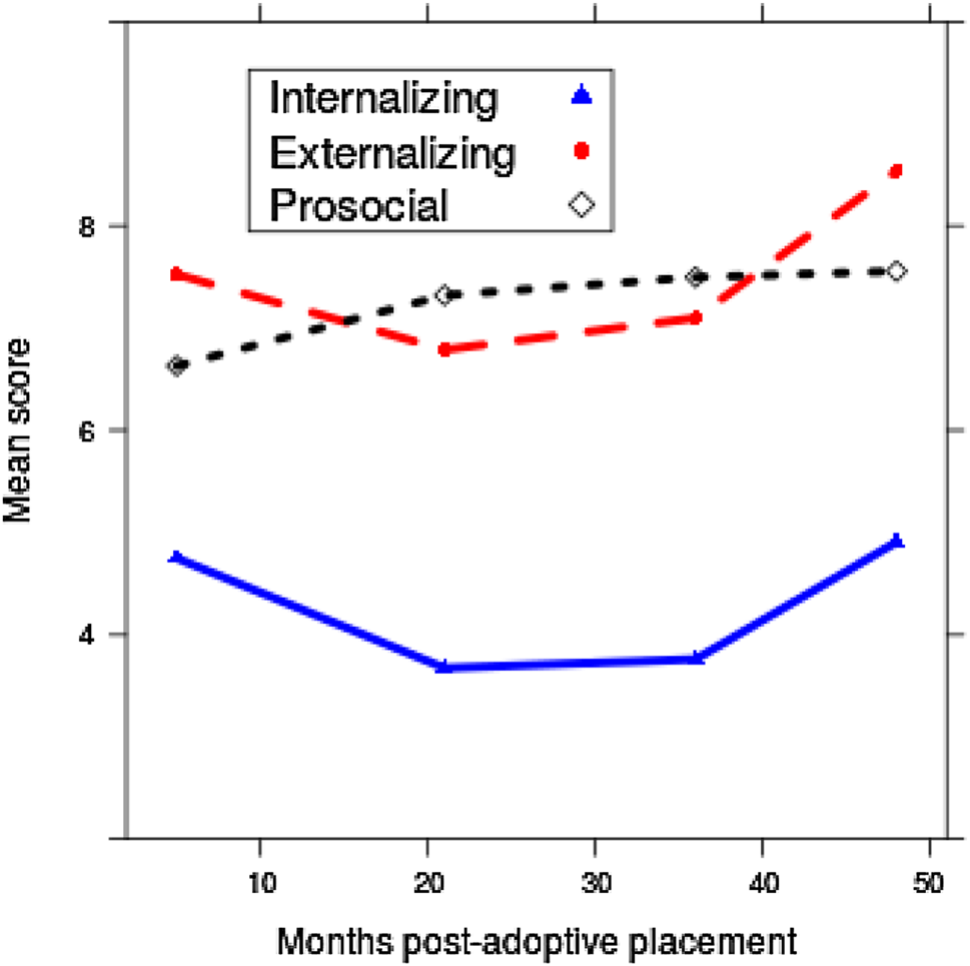
Sample means for internalizing and externalizing problems, and prosocial behavior for each wave of the study

Figure 1 shows the mean value of each outcome at each wave of the study. Children’s internalizing and externalizing scores were higher than the general population, see [9], and remained relatively stable over time; *t* tests comparing internalizing and externalizing scores at Waves 1 and 4 (5 months and 48 months, respectively) were not significant (internalizing *M* = 4.75 at W1, *M* = 4.91 at W4, *p* = 0.80; externalizing *M* = 7.52 at W1, *M* = 8.54 at W4, *p* = 0.10). Across the sample and at each wave, parents reported more externalizing than internalizing problems (all *p*s < 0.01). Prosocial behavior scores increased from 5 to 48 months post-placement *M* = 6.63 at W1, *M* = 7.56 at W4, *p* = 0.01).

Descriptive statistics for all study predictors and covariates of interest are presented in Table 1. Bivariate associations between predictors and covariates of interest and internalizing and externalizing problems pooled across all four waves are presented in Table 2. To clarify, this correlation matrix pools observations across all four waves, post-imputation. We observed strong correlations between ACEs and number of moves, days with birth parents, and in care. Associations between age at placement, child age at the commencement of the study, and number of days with birth parents were particularly high *r*s = 0.92–0.95; therefore of these three variables, only the number of days with birth parents was included in the subsequent models to avoid issues of multicollinearity.

**Table 1:** Summary statistics for variables of interest

Variable	
Number of moves	
Median (range)	1 (0–13)
Number of adverse childhood experiences	
Median (range)	2 (1–9)
Number of days with birth parents	
Mean (SD)	522.92 (611.74)
Range	0–2344
Number of days in care	
Mean (SD)	537.09 (285.74)
Range	203–1401
Age at placement	
Mean (SD)	2.36 (2.20)
Range	0–9
Child gender *n* (%) female	47 (49.0)
Siblings in household *n* (%) with sibling	43 (44.8)
Adoptive parent age at the time of adoption	
Mean (SD)	40.67 (6.98)
Range	22–62
Adoptive parent relationship status *n* (%) couple adopter	84 (87.5)
Adoptive parent education *n* (%) postgraduate or higher degree	35 (36.5)
Adoptive parent employment *n* (%) full time or part time paid	72 (75.0)
Adoptive parent income *n* (%)	
Up to £19,999	11 (11.5)
£20,000 to 49,999	42 (43.8)
£50,000 and over	43 (44.8)

**Table 2:** Correlation matrix of variables of interest in the present study

	1	2	3	4	5	6	7	8	9	10	11	12	13	14	15	16
1. Number of moves	–															
2. ACE count	0.43∗	–														
3. Days with birth parents	0.14∗	0.68∗	–													
4. Days in care	0.06	0.40∗	0.39∗	–												
5. Age at placement	0.13∗	0.66∗	0.92∗	0.68∗	–											
6. Resp. age^^^	− 0.21∗	0.12∗	0.20∗	0.38∗	0.30∗	–										
7. Child gender	− 0.05	0.00	0.11	− 0.13∗	0.03∗	− 0.17	–									
8. Child age^^^	0.14∗	0.67∗	0.92∗	0.65∗	0.95∗	0.30∗	0.04	–								
9. Child sibling (Y/N)^^^	0.19∗	0.16∗	0.14∗	0.01	0.13∗	− 0.08	− 0.06	0.09∗	–							
10. Resp. marital status^^^	0.03	− 0.05	− 0.06	− 0.45∗	− 0.22∗	− 0.29∗	0.10	− 0.20∗	0.09	–						
11. Resp. education^^^	− 0.17∗	− .14∗	− 0.09	− 0.11	− 0.12∗	0.10	− 0.19∗	− 0.13∗	0.06	− 0.04	–					
12. Resp. income^^^	− 0.07	0.03	0.00	− 0.29∗	− 0.12∗	− 0.08	− 0.03	− 0.10	0.13∗	0.36∗	0.06	–				
13. Resp. employment^	0.02	− 0.01	− 0.02	− 0.05	− 0.06	− 0.07	− 0.10	− 0.01	− 0.11∗	0.00	0.04	0.11∗	–			
14. Internalizing problems	0.01	0.23∗	0.27∗	0.19∗	0.30∗	0.15∗	− 0.16∗	0.24∗	0.24∗	− 0.18∗	0.09	− 0.04	− 0.12∗	–		
15. Externalizing problems	− 0.05	0.21∗	0.28∗	0.15∗	0.28∗	0.14∗	− 0.14∗	0.26∗	0.12∗	− 0.08	− 0.12∗	0.05	− 0.05	0.56∗	–	
16. Prosocial behavior	0.08	0.14∗	0.13∗	0.02	0.09	− 0.07	0.30∗	0.14∗	− 0.13∗	0.16∗	0.05	− 0.05	0.17∗	− 0.29∗	− 0.39∗	–

*p < 0.05

^^^At wave 1. The correlation coefficients were generated using an imputed dataset. Internalizing and externalizing problems, and prosocial behaviors are pooled across all four waves of the study. Child sibling was coded as 1 = yes, 0 = no and gender as 1 = male, 2 = female

### Adoptees’ internalizing problems

Number of moves and ACEs consistently predicted adoptees’ internalizing problems; see estimated coefficients in Table 3. Number of ACEs were positively associated with children’s internalizing problems; number of moves was negatively associated with internalizing problems in the autoregressive models. The substantive effect of an additional ACE was conditional on the model. An additional adverse experience was associated with a 0.15 and 0.21 increase in internalizing problems in the AR-1 and 4-wave AR-1 models, respectively (*p*s < 0.05); although not statistically significant, the fixed-effects model showed coefficients in the same and expected direction. An additional move was associated with a 0.17 decrease in internalizing problems in the 4-wave AR-1 model (*p* < 0.01). A one-unit increase in days in care was associated with a 0.001 decrease in internalizing problems in both the AR-1 and 4-wave AR-1 models (all *p*s < 0.05, Table 3). According to the 4-wave AR-1 model, boys had more internalizing problems over time (coeff. = − 1.00, *p* < 0.05).

**Table 3:** Estimated coefficients for associations between predictor variables and internalizing and externalizing problems, and prosocial behavior

	Internalizing problems			Externalizing problems			Prosocial behavior		
	FE	AR-1	AR-1 (4 Waves)	FE	AR-1	AR-1 (4 Waves)	FE	AR-1	AR-1 (4 Waves)
Number of moves	− 0.107 (0.334)	− 0.069 (0.055)	− 0.167** (0.033)	− 0.547 (0.327)	− 0.291** (0.054)	− 0.302** (0.104)	0.103 (0.259)	0.015 (0.033)	0.023 (0.067)
ACE count	0.591 (0.453)	0.152* (0.076)	0.211** (0.075)	− 0.153 (0.288)	0.238** (0.040)	0.273** (0.099)	0.120 (0.213)	0.025 (0.027)	0.009 (0.032)
Days in care		− 0.001* (0.0003)	− 0.001** (0.0001)		− 0.001** (0.0004)	− 0.001 (0.001)		0.001** (0.0001)	0.001** (0.0003)
Days with birth parents		0.001 (0.0005)	0.001 (0.0004)		0.0003** (0.0001)	− 0.00005 (0.0001)		0.0002 (0.0003)	0.0002 (0.0002)
Child gender		− 0.689 (0.577)	− 0.998** (0.466)		− 0.876** (0.303)	− 0.872 (0.609)		0.943** (0.095)	0.940** (0.287)
Internalizing problems_*t−*1_		0.408* (0.196)	0.435* (0.182)						
Externalizing problems_*t−*1_					0.456** (0.156)	0.443** (0.169)			
Prosocial behavior_*t−*1_								0.389** (0.076)	0.431** (0.148)
Intercept	3.360** (1.010)	5.100 (2.720)	8.080** (1.340)	6.750** (1.020)	5.540** (1.610)	7.790** (0.664)	5.45** (0.781)	2.090 (1.250)	1.090 (1.030)
Observations	318	222	177	318	222	177	318	222	177
Adjusted *R*^2^	0.376	0.292	0.323	0.498	0.302	0.261	0.453	0.384	0.401
*F* statistic	2.910**	8.020**	7.460**	4.140**	8.350**	5.790**	3.620**	11.600**	10.100**

FE = fixed-effects model, AR-1 = autoregressive model, AR-1 (4 waves) = autoregressive model for respondents who participated in all four waves. Coefficients are unstandardized to allow for direct interpretability. Standard errors are clustered by individual respondent and presented in brackets below the coefficients. Models are adjusted for respondent (adoptive parent) age at adoption, relationship status, education, income, employment, and siblings in household (see supplementary materials for all coefficients)

*p < 0.05, **p < 0.01

### Adoptees’ externalizing problems

In the autoregressive models, number of moves were negatively associated with children’s externalizing problems. A one-unit increase in the number of moves was associated with a 0.29 decrease in externalizing problems in the AR-1 model and a 0.30 decrease in the 4-wave AR-1 model (all *p*s < 0.01). The relationship between number of ACEs and children’s externalizing problems was in the expected direction, but only statistically significant in the autoregressive models. A one-unit increase in number of ACEs increased externalizing problems by 0.24 and 0.27 in the AR-1 and four-wave models, respectively (*p*s < 0.01, Table 3). According to the AR-1 model, a one-unit increase in days in care was associated with a 0.001 decrease in externalizing problems, and a one-unit increase in days with birth parents was associated with a 0.0003 increase in externalizing problems (both *p*s < 0.01). The AR-1 model indicated that boys had more externalizing problems over time (coeff. = – 0.88, *p* < 0.01).

### Adoptees’ prosocial behavior

In the AR models, girls were more prosocial over time (coeff. = 0.94, *p* < 0.01 in AR-1 and 4-wave AR-1). The number of days in care was associated with an increase in prosocial behavior over time; a one-unit increase in days in care was associated with a 0.001 increase in prosocial behavior in the AR-1 and 4-wave AR-1 models (all *p*s < 0.01).

### Control variable effects

There were no respondent control variables that were consistently associated with the internalizing and externalizing problems. Employed respondents reported adoptive children with 0.96 fewer internalizing problems (AR-1, an effect which was larger in the 4-wave model), and single adopters and adopters who had postgraduate or higher degrees reported that their children had more internalizing problems according to the 4-wave AR-1 models (coeffs. = – 2.00 and 0.36, respectively, *p*s < 0.05). Couple adopters, those with postgraduate or higher degrees, or those who earned £20,000 to £49,999 compared to £19,999 or lower, reported their child to have fewer externalizing problems (AR-1 coeffs. = – 0.62, – 1.25, and – 0.42, respectively, all *p*s < 0.05, with similar and significant effects in the 4-wave AR-1 models). In the prosocial model, several estimated coefficients were statistically significant. Married or cohabiting adopters reported more prosocial behavior (coeff. = 1.66, *p* < 0.01 in the AR-1 model, corroborated by the 4-wave AR-1 model), as did respondents with postgraduate or higher degrees (coeff. = 0.54, *p* < 0.01 in AR-1 model), and those in employment (coeff. = 1.02, *p* < 0.01 in AR-1 model and similarly in the 4-wave AR-1 model). By contrast, increased respondent income was associated with fewer prosocial behaviors according to the AR-1 model (coeff. = – 0.54, *p* < 0.01) (see supplementary materials for all models).

### Exploratory analysis

The unexpected negative effects of the number of moves and number of days in care on internalizing and externalizing in the AR-1 models behaviors merited closer attention, therefore we explored possible interactive effects between predictors of interest. We suspected that these variables may mitigate or condition the effects of each other and warranted further analysis. We interacted number of moves with number of ACEs, days in care, or with days with birth parents, and we interacted days in care with number of ACEs or with days with birth parents. This was done to determine under what conditions the effects we observed from the number of moves and number days in care were mitigated or exacerbated by other key variables of interest. Additionally, this enabled us to pinpoint the conditions under which these effects were meaningful: for example, the conditional effect of moving children as their time in care increased or decreased.

**Fig. 2:**
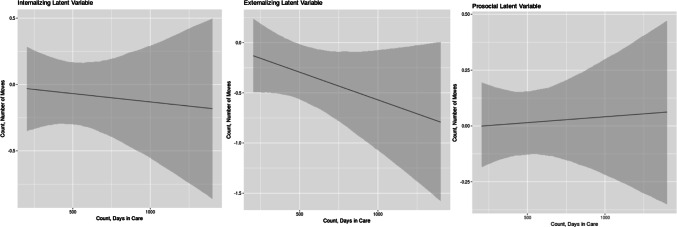
Marginal effects, number of moves across range of days in care for internalizing and externalizing problems and prosocial behavior

We found a significant interactive effect between the number of moves and days in care on externalizing problems (coeff. = − 0.001, *p* < 0.01, see Table 4); only under specific circumstances did the number of moves and days in care reduce externalizing behaviors. Specifically, moving a child within the care system did not reduce externalizing problems unless the child was in the care system for less than approximately 400 days. For children in the care system after approximately 400 days, there was no change in externalizing problems as a function of moves. We report marginal-effects plots demonstrating these interactions in Fig. 2 (AR-1 models; see the supplementary materials for marginal-effects plots for four-wave AR-1 models).

**Table 4:** Estimated coefficients for associations between predictor variables and internalizing and externalizing problems, and prosocial behavior

	Number of moves × days in care models			ACE count × days in care models		
	Internalizing	Externalizing	Prosocial	Internalizing	Externalizing	Prosocial
Number of moves	− 0.197 (0.135)	0.017 (0.151)	− 0.021 (0.166)	− 0.176** (0.066)	− 0.329* (0.145)	0.044** (0.006)
ACE count	0.163* (0.076)	0.260** (0.017)	0.009 (0.027)	0.213** (0.056)	0.507* (0.221)	− 0.164** (0.034)
Days in care	− 0.0005** (0.0001)	0.0002 (0.0002)	0.001 (0.001)	− 0.0001 (0.001)	0.001 (0.002)	− 0.0003 (0.0003)
Days with birth parents	0.001 (0.0005)	0.0003** (0.00004)	0.0001 (0.0002)	0.001* (0.0004)	0.0003** (0.0001)	0.0001 (0.0002)
Child gender	− 0.754 (0.711)	0.878 (0.624)	0.946** (0.282)	− 0.733 (0.730)	− 0.941 (0.548)	0.937** (0.279)
Internalizing problems_*t−*1_	0.434* (0.185)			0.435* (0.188)		
Externalizing problems_*t−*1_		0.444** (0.136)			0.464** (0.140)	
Prosocial behavior_*t−*1_			0.403** (0.069)			0.399** (0.059)
Number of moves × days in care	0.00005 (0.0002)	− 0.001** (0.0001)	0.0001 (0.0003)			
ACE count × days in care				− 0.0001 (0.0002)	− 0.0005 (0.0004)	0.0003** (0.0001)
Intercept	5.380* (2.700)	5.100** (1.070)	1.970 (1.760)	5.070 (3.080)	4.750**(1.040)	2.660 (1.490)
Observations	222	222	222	222	222	222
Adjusted *R*^2^	0.310	0.296	0.380	0.310	0.296	0.391
*F* statistic	8.090**	7.630**	10.700**	8.100**	7.620**	11.200**

AR-1 autoregressive models. Coefficients are unstandardized to allow for direct interpretability. Standard errors are clustered by individual respondent and presented in brackets below the coefficients. Models are adjusted for respondent (adoptive parent) age at adoption, relationship status, education, income, employment, and siblings in household (see online supplement for all coefficients)

*p < 0.05, **p < 0.01

**Fig. 3:**
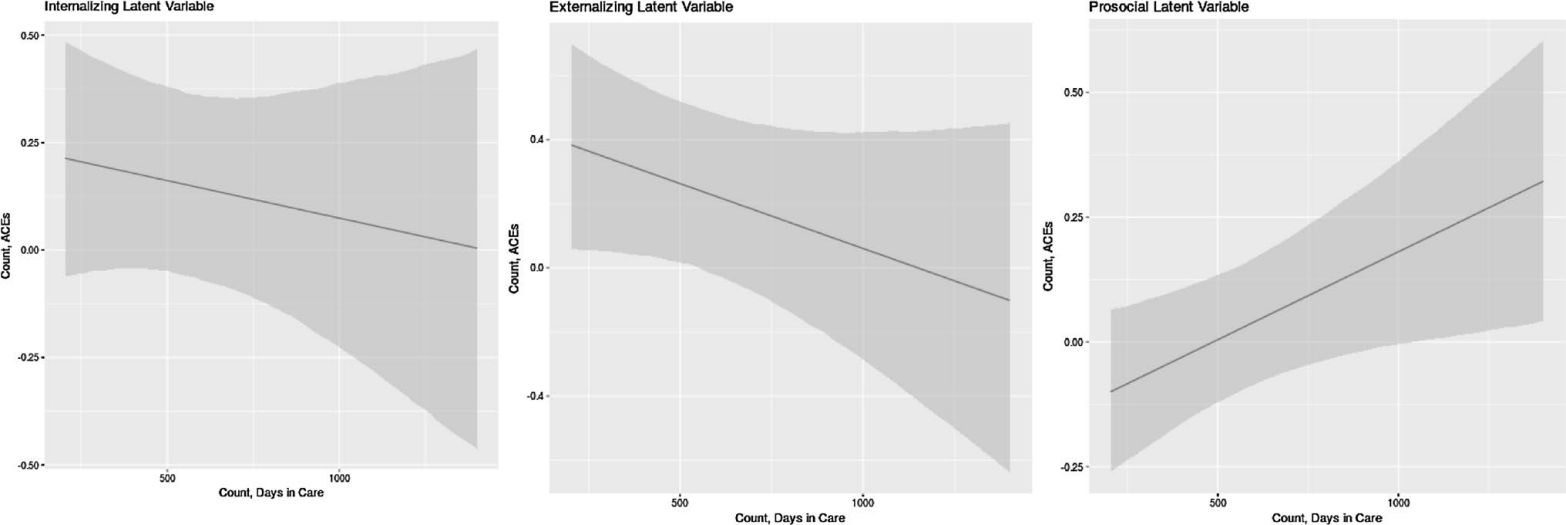
Marginal effects, number of ACEs across range of days in care for internalizing and externalizing problems and prosocial behavior

Other interactions yielded interesting associations (see Table 4). The effect of ACEs on externalizing behaviors was mitigated by time spent in care; following 550 days in care, the long-term effects of ACEs ceased to have a statistically significant effect on externalizing problems (see Fig. 3 for marginal-effects plots demonstrating interactions in AR-1 models). After that length of time, our model can say little more about the effects of ACEs on children’s mental health. We found some evidence that long-term time in care can improve the prosocial behavior of children with more ACEs (coeff. = 0.0003, *p* < 0.01), yet after 1100 days in care, the effect of ACEs on prosocial behavior was statistically significant (Fig. 3). Four-wave AR-1 marginal-effects plots are presented in the supplementary materials.

## Discussion

This study investigated the mental health of children adopted from public care in the UK. We did not discern improvement in adopted children’s mental health over 4 years post-placement, underscoring the need for a better understanding of pre-adoptive factors that predict children’s long-term mental health problems. We used fixed-effects models and autoregressive distributive lag models to estimate coefficients exploring the effects of multiple interrelated risk factors on children’s internalizing and externalizing problems and prosocial behavior.

We identified a persistent negative impact of ACEs on adopted children’s internalizing and externalizing scores over time. Given that children adopted from care in the UK have a high prevalence of ACEs [9] that impact on adult psychological and physical health [11, 13], our findings have relevance for prevention and intervention strategies by underscoring the need to protect children from accumulating ACEs in early childhood, and for effective interventions once ACEs are identified.

Exploratory analyses of negative associations between number of days in care and children’s problem scales showed that, following approximately 550 days in care, the long-term effects of ACEs on externalizing problems were no longer significant. This suggests a positive mitigating effect of time with foster families for children who have experienced more pre-placement adversity such as abuse, neglect, and household dysfunction. Our results attest to the value of having as much relevant biographical information about a child’s life before and during care as possible and for this to be shared with foster carers (pre-adoptive placement) and adopters in a professional and judicious manner. In this way, our findings support recent recommendations and new service developments (in the UK) which emphasize soliciting information and views from everyone involved in the care of children pre- and post-adoption with regard to their psychological development and experiences before and in care [28, 29]. This may make an important contribution to parents’ capacity to understand, anticipate, adapt, and respond to children’s needs and behaviors after placement. Adoptive families should be encouraged to seek support early, via mainstream statutory services (e.g. mental health in schools; health visiting). However, data on effective strategies are limited [13], and identification of mechanisms whereby ACEs impact children’s mental health may inform such strategies [30, 31].

Our findings extend earlier work by investigating adopted children’s prosocial behavior. In the present study, children who spent more days in care had slightly higher prosocial behavior scores, although these effects are small. We found that time in care mitigated the negative effect of an additional ACE on prosocial behavior, but this did not hold if children were in care long term. Possibly, these findings are driven by a child’s removal into a safe, secure, and positive family setting, underscoring the importance of consistent, sensitive early parenting that models and reinforces sympathetic behavior and empathetic concern known to promote prosocial tendencies [32]. Given that some types of early prosocial behaviors protect against later risk for mental health problems [33], the pathways by which time in care mitigates the impact of adversity on adoptees’ prosocial behavior warrants further study.

Contrary to our hypothesis, we found negative associations between the number of moves and children’s problem scales. In exploratory analyses, we found that moving children in care from one home to another only provides benefits in rare circumstances, whereby the benefits associated with moving children in care were limited to those who had spent less than approximately 400 days in care. Given that earlier work demonstrates that multiple moves have a negative impact on a child’s adjustment [15, 16], this finding warrants further investigation. Firstly, what constitutes a change in placement must be clearly defined; although some studies include short-term stays (e.g. 1 or 2 days in hospital; [15]), in the present study we investigated removals deemed significant by the child’s social worker. Secondly, the nature of the moves must be considered further. Some moves may have included stays in kinship care rather than a placement with ‘strangers’, providing children with a better sense of stability and opportunity to remain in their existing network. Our findings should be taken tentatively, as the beneficial conditions under which moving the children in this sample were rare and moves should be conducted with considerable caution.

This study has limitations. Our aim in this study was to disentangle the impact of various pre-adoptive experiences on child adjustment; however, given that prenatal substance abuse is associated with adoptees’ mental health problems [34], the long-term effects of exposure in utero should also be considered in future studies. Although investigation of ACEs via prospective and independent reporting from social workers is a major strength of this study—by reducing a common issue of response bias associated with retrospective self-reporting in ACEs studies [35]—the investigation of cumulative ACEs leads to the statistical treatment of all childhood adversities as equal in their impact on child functioning [36]. However, recent studies indicate that specific ACE categories incur varying levels of risk [37]. Therefore, future work must consider how ACEs are weighted and how they cluster together [36]. Our findings are also limited by having a single respondent report on each child in the study post-placement, which may distort magnitudes of associations due to shared method variance [38]. Finally, it is worthy of note that the families in the present study—in common with other families in the target population—were generally of high SES.

Our exploratory interactive models models cannot determine conclusively whether the preplacement risk factors mitigate each other. It is possible that a three-way interaction between ACEs, number of moves, and number of days in care exists, but to determine whether this is the case, additional waves of this study and replication in larger longitudinal samples is needed. Our exploratory findings show very specific conditions under which these predictors influence adoptees’ outcomes and should not be taken as prescriptive. Instead, we show that our counterintuitive findings are conditioned by a variety of measures, and that research is required to determine how early experiences in birth families and interventions of varying duration by the state shape the development of children who spend time in local authority care.

Children adopted from care are more likely to experience enduring mental health problems that persist in the years following their adoptive placement. Determining the risk factors for adoptees’ enduring mental health problems is vital to inform early interventions to improve children’s outcomes and for the prevention of adoptive family crisis and breakdown. Given the deleterious effects of early adversity on health and functioning in later life [13] and the vulnerability of children adopted from care [9], we highlight that identification, careful documentation, and transparency in sharing children’s histories of early adversity among caregivers are potentially powerful tools for identifying children at risk for enduring mental health problems and a target for intervention.

## Electronic supplementary material

Below is the link to the electronic supplementary material.

Supplementary file1 (DOCX 205 kb)
