# Supplementary material for: Early adversity predicts adoptees’ enduring emotional and behavioral problems in childhood
Source: Eur Child Adolesc Psychiatry. 2020 May 28;30(5):721–32. doi: 10.1007/s00787-020-01553-0 (PMC8060221; doi:10.1007/s00787-020-01553-0)
Supplement: Supplementary file 1 — Supplementary file1 (DOCX 205 kb) [file 787_2020_1553_MOESM1_ESM.docx]

**Online Supplementary Appendix**

**Early Adversity Predicts Adoptees’ Enduring Emotional and Behavioral Problems in Childhood**

***European Child and Adolescent Psychiatry***

Amy L. Paine^1^

Kevin Fahey^2^

Rebecca E. Anthony^3^

Katherine H. Shelton^1^

^1^School of Psychology, Cardiff University, Tower Building, 70 Park Place, Cardiff, CF10 3AT, UK.

^2^Department of Political and Cultural Studies, Swansea University, James Callaghan Building, Sketty, Swansea, SA2 8PZ, UK.

^3^Centre for the Development and Evaluation of Complex Interventions for Public Health Improvement (DECIPHer), School of Social Sciences, Cardiff University, 1-3 Museum Place, Cardiff, CF10 3BD, UK.

Correspondence to Dr. Amy L. Paine at [paineal@cardiff.ac.uk](mailto:paineal@cardiff.ac.uk)

**Online Supplementary Appendix**

**Data**

We show distributions of outcome measures after construction in Figure A1.


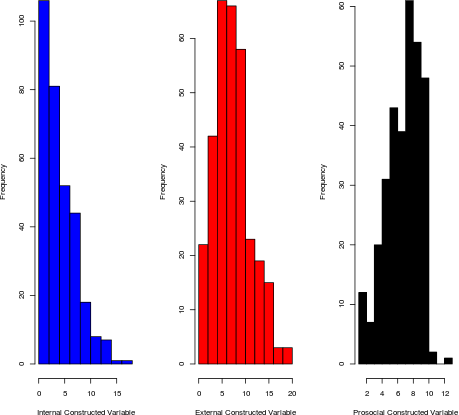


Figure A1. *Distribution of outcome variables after construction*

**Analysis**

**Fixed effects, autoregressive, and four-wave autoregressive models.** Table A1 shows the coefficients for the full internalizing symptoms, externalizing problems, and prosocial behavior models (See Table 3 in manuscript).

Table A1

*Estimated coefficients for associations between predictor variables and internalizing and externalizing problems, and prosocial behavior.*

|  | **Internalizing problems** | | | **Externalizing problems** | | | **Prosocial behavior** | | |
| --- | --- | --- | --- | --- | --- | --- | --- | --- | --- |
|  | FE | AR-1 | AR-1 (4 Waves) | FE | AR-1 | AR-1 (4 Waves) | FE | AR-1 | AR-1 (4 Waves) |
| Number of moves | -0.107  (0.334) | -0.069  (0.055) | -0.167^**^  (0.033) | -0.547  (0.327) | -0.291^**^  (0.054) | -0.302^**^  (0.104) | 0.103  (0.259) | 0.015  (0.033) | 0.023  (0.067) |
| ACE count | 0.591  (0.453) | 0.152^*^  (0.076) | 0.211^**^  (0.075) | -0.153  (0.288) | 0.238^**^  (0.040) | 0.273^**^  (0.099) | 0.120  (0.213) | 0.025  (0.027) | 0.009  (0.032) |
| Days in care |  | -0.001^*^  (0.0003) | -0.001^**^  (0.0001) |  | -0.001^**^  (0.0004) | -0.001  (0.001) |  | 0.001^**^  (0.0001) | 0.001^**^  (0.0003) |
| Days with birth parents |  | 0.001  (0.0005) | 0.001  (0.0004) |  | 0.0003^**^  (0.0001) | -0.00005  (0.0001) |  | 0.0002  (0.0003) | 0.0002  (0.0002) |
| Child gender |  | -0.689  (0.577) | -0.998^**^  (0.466) |  | -0.876^**^  (0.303) | -0.872  (0.609) |  | 0.943^**^  (0.095) | 0.940^**^  (0.287) |
| Respondent age |  | -0.012  (0.030) | -0.047^**^  (0.014) |  | 0.028  (0.043) | 0.012  (0.035) |  | -0.017  (0.012) | -0.004  (0.013) |
| Respondent relationship status |  | -1.630  (1.080) | -2.000^*^  (0.995) |  | -0.617^**^  (0.086) | -0.846^**^  (0.040) |  | 1.660^**^  (0.500) | 1.620^**^  (0.415) |
| Respondent education |  | 0.267  (0.193) | 0.356^**^  (0.076) |  | -1.250^**^  (0.475) | -1.060^*^  (0.510) |  | 0.538­^*‑^  (0.265) | 0.552  (0.396) |
| Respondent income (2) |  | -0.663  (0.993) | -1.380  (1.220) |  | -0.424^**^  (0.116) | -1.320  (0.711) |  | -0.199  (0.140) | -0.001  (0.444) |
| Respondent income (3) |  | -0.348  (1.010) | -0.946  (1.250) |  | 0.069  (0.123) | -0.852  (0.823) |  | -0.540^**^  (0.129) | -0.508  (0.335) |
| Respondent employment |  | -0.955^*^  (0.388) | -1.340^**^  (0.391) |  | -0.714  (0.830) | -1.040  (0.945) |  | 1.020^**^  (0.187) | 1.030^**^  (0.236) |
| Sibling(s) in household | -2.880  (4.120) | 0.727^**^  (0.163) | 0.767^**^  0.069) | 4.530  (2.800) | 0.284  (0.293) | 0.261  (0.420) | 1.280  (2.010) | -0.034  (0.173) | 0.007  (0.153) |
| Internalizing problems *_t_* _- 1_ |  | 0.408^*^  (0.196) | 0.435^*^  (0.182) |  |  |  |  |  |  |
| Externalizing problems *_t_* _- 1_ |  |  |  |  | 0.456^**^  (0.156) | 0.443^**^  (0.169) |  |  |  |
| Prosocial behavior *_t_* _- 1_ |  |  |  |  |  |  |  | 0.389^**^  (0.076) | 0.431^**^  (0.148) |
| Intercept | 3.360^**^  (1.010) | 5.100  (2.720) | 8.080^**^  (1.340) | 6.750^**^  (1.020) | 5.540^**^  (1.610) | 7.790^**^  (0.664) | 5.45^**^  (0.781) | 2.090  (1.250) | 1.090  (1.030) |
| Observations | 318 | 222 | 177 | 318 | 222 | 177 | 318 | 222 | 177 |
| Adjusted *R*^2^ | 0.376 | 0.292 | 0.323 | 0.498 | 0.302 | 0.261 | 0.453 | 0.384 | 0.401 |
| *F* statistic | 2.910^**^ | 8.020^**^ | 7.460^**^ | 4.140^**^ | 8.350^**^ | 5.790^**^ | 3.620^**^ | 11.600^**^ | 10.100^**^ |

*Note.* **p*<.05, ***p*<.01.

**Multiple Imputation.**

Some individuals exhibited missingness in the data. We demonstrate the availability of data in both long and wide formats below. The long format shows each unique observation irrespective of time period, while the wide format shows each unique respondent and missingness for each covariate at each time period (see Figure A2).

**
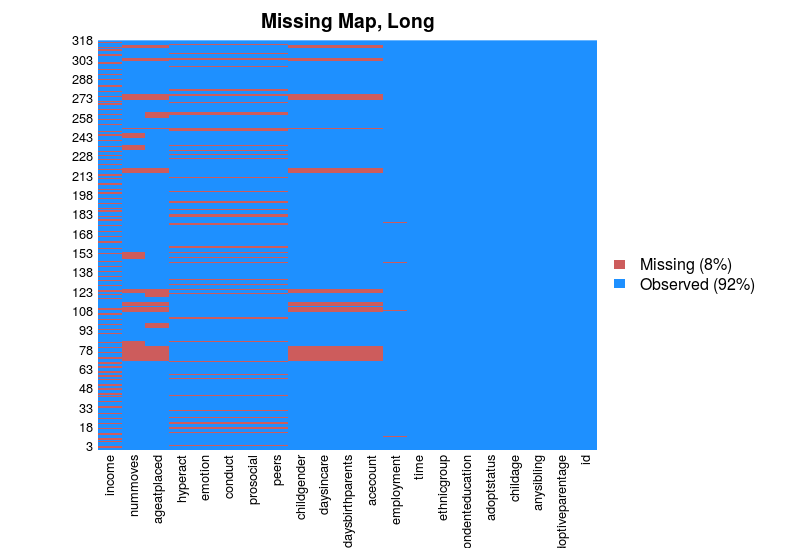
**

**
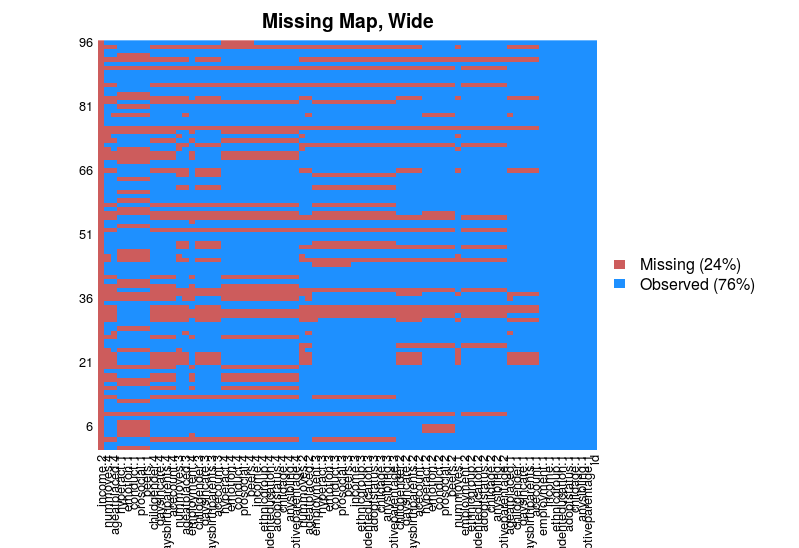
**

Figure A2. *Missing data maps.*

To address this problem, we generated 1000 datasets that used multiple imputation to address missingness in the data. We estimated the AR-1 models for each of the 1000 datasets, obtained the estimated coefficients for the pre-adoptive risk variables (a focus on ACEs and number of moves), and plotted associated *t*-statistics in Figure A3. These statistics show the number of imputed datasets, to the right of the black line, where the estimated coefficients are statistically significant.


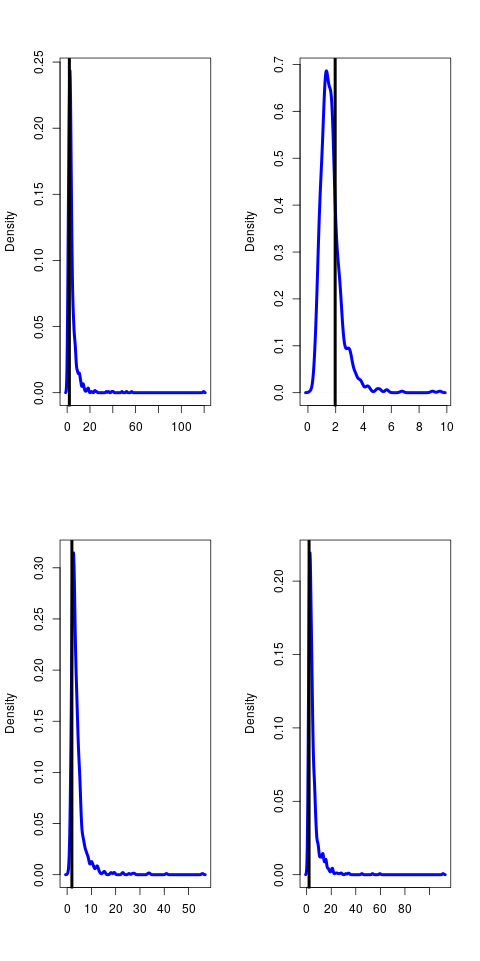


Figure A3. *1000 imputed datasets: t statistics for number of moves (left) and ACEs (right), in AR-1 internalizing model (top) and externalizing model (bottom).*

These plots demonstrate that in most cases, the selected imputed dataset produces similar, if not identical, results to those reported in the manuscript. The one exception is that the coefficient for ACEs in the internalizing model was dependent on which imputed dataset was used; in approximately half of the imputed datasets, the *t*-statistic was less than 1.96 (although the coefficient was still positive in the remaining cases).

**Listwise deletion, multiple imputation, and re-estimation.** As a robustness check, we re-estimated coefficients from our AR-1 model using listwise deletion rather than multiple imputation for all observations and all variables of missingness. This significantly truncates the number of available observations. Those results are reported in Table A2. Additionally, we re-estimated coefficients from our AR-1 models that use imputation save for the outcome variables, which were not used to inform the imputation process. Those results are reported in Table A3.

In Table A2, coefficients are in the expected direction and statistically significant (save for the externalizing model ACE count variable, although it is in the expected direction. We concluded that multiple imputation, preferable to listwise deletion, does not meaningfully alter the results nor increase the likelihood of a Type I error.

Table A2

*Estimated coefficients, listwise deletion instead of multiple imputation. AR-1 models.*

|  | Internalizing | Externalizing | Prosocial |
| --- | --- | --- | --- |
| Number of moves | -0.138^*^ | -0.155^**^ | 0.036 |
|  | (0.064) | (0.056) | (0.038) |
| ACE count | 0.341^**^ | 0.295 | -0.080 |
|  | (0.063) | (0.170) | (0.098) |
| Days with birth parents | -0.0001 | -0.0002 | 0.00000 |
|  | (0.001) | (0.001) | (0.001) |
| Days in care | -0.001^**^ | -0.001 | 0.001^**^ |
|  | (0.0003) | (0.001) | (0.00002) |
| Respondent age | -0.052 | -0.022 | -0.016 |
|  | (0.031) | (0.041) | (0.087) |
| Child gender | -0.963 | -0.616 | 0.622^**^ |
|  | (0.954) | (0.977) | (0.103) |
| Sibling(s) in household | 0.535^**^ | 0.519 | 0.099^**^ |
|  | (0.156) | (0.431) | (0.026) |
| Respondent relationship status | -2.920 | -1.150^**^ | 1.380^**^ |
|  | (2.070) | (0.445) | (0.320) |
| Respondent education | 0.580^**^ | -0.449 | 0.014 |
|  | (0.039) | (0.380) | (0.019) |
| Respondent income (2) | -0.151 | 0.339 | 0.316 |
|  | (2.290) | (0.228) | (0.281) |
| Respondent income (3) | 0.417 | 1.200^**^ | -0.659^**^ |
|  | (2.210) | (0.169) | (0.170) |
| Respondent employment | -1.290^*^ | -2.010^**^ | 0.969^**^ |
|  | (0.650) | (0.198) | (0.295) |
| Internalizing*_t-1_* | 0.609^**^ |  |  |
|  | (0.117) |  |  |
| Externalizing *_t-1_* |  | 0.585^**^ |  |
|  |  | (0.106) |  |
| Prosocial *_t-1_* |  |  | 0.509^**^ |
|  |  |  | (0.139) |
| Intercept | 7.840^**^ | 7.160^**^ | 2.150 |
|  | (1.530) | (1.640) | (5.240) |
| Observations | 109 | 109 | 109 |
| Adjusted *R*^2^ | 0.406 | 0.357 | 0.436 |
| *F* statistic | 6.680^**^ | 5.610^**^ | 7.420^**^ |

*Note.* * *p*<.05, ***p*<.01.

Table A3

*Multiple imputation, outcome variables excluded from imputation. AR-1 models.*

|  | Internalizing | Externalizing | Prosocial |
| --- | --- | --- | --- |
| Number of moves | -0.111 | -0.213^**^ | 0.003 |
|  | (0.066) | (0.022) | (0.023) |
| ACE count | 0.142^*^ | 0.224^**^ | -0.004 |
|  | (0.063) | (0.051) | (0.039) |
| Days with birth parents | 0.001 | 0.00003 | 0.00001 |
|  | (0.0003) | (0.0003) | (0.0002) |
| Days in care | -0.001^*^ | -0.002^**^ | 0.001^*^ |
|  | (0.0003) | (0.0004) | (0.0003) |
| Respondent age | -0.034 | 0.014 | -0.020 |
|  | (0.044) | (0.060) | (0.022) |
| Child gender | -0.700 | -0.823 | 0.615^**^ |
|  | (0.456) | (0.511) | (0.212) |
| Sibling(s) in household | 0.584^*^ | -0.074 | 0.049 |
|  | (0.238) | (0.768) | (0.068) |
| Respondent relationship status | -1.350 | -0.391 | 1.090^*^ |
|  | (1.440) | (0.326) | (0.430) |
| Respondent education | 0.133 | -0.843^**^ | 0.171 |
|  | (0.264) | (0.312) | (0.166) |
| Respondent income (2) | -0.454 | -0.200 | -0.180 |
|  | (0.915) | (0.440) | (0.252) |
| Respondent income (3) | -0.407 | -0.009 | -0.198 |
|  | (1.030) | (0.305) | (0.419) |
| Respondent employment | -1.440^**^ | -1.140 | 0.854^**^ |
|  | (0.102) | (0.653) | (0.099) |
| Internalizing*_t-1_* | 0.506^*^ |  |  |
|  | (0.210) |  |  |
| Externalizing *_t-1_* |  | 0.587^**^ |  |
|  |  | (0.126) |  |
| Prosocial *_t-1_* |  |  | 0.522^**^ |
|  |  |  | (0.072) |
| Intercept | 6.180^**^ | 5.720^**^ | 2.390 |
|  | (1.920) | (2.190) | (1.610) |
| Observations | 182 | 182 | 182 |
| Adjusted *R*^2^ | 0.331 | 0.366 | 0.442 |
| *F* statistic | 7.880^**^ | 9.050^**^ | 12.000^**^ |

*Note.* * *p*<.05, ***p*<.01.

**Interactions between days in care and long term risk factors**

The tables associated with the marginal effects plots reported in the manuscript are presented in Table A4. Those models using AR-1 (four wave) subset are reported in Table A5.

Table A4

*Interactions between Days in Care and long-term risk factors. AR-1 (All-Waves) models used. Standard errors reported in parentheses (see Table 4 in manuscript)*

|  | Number of moves x days in care models | | | ACE count x days in care models | | |
| --- | --- | --- | --- | --- | --- | --- |
|  | Internalizing | Externalizing | Prosocial | Internalizing | Externalizing | Prosocial |
| Number of moves | -0.197 | 0.017 | -0.021 | -0.176^**^ | -0.329^*^ | 0.044^**^ |
|  | (0.135) | (0.151) | (0.166) | (0.066) | (0.145) | (0.006) |
| Days in care | -0.0005^**^ | 0.0002 | 0.001 | -0.0001 | 0.001 | -0.0003 |
|  | (0.0001) | (0.0002) | (0.001) | (0.001) | (0.002) | (0.0003) |
| ACE count | 0.163^*^ | 0.260^**^ | 0.009 | 0.213^**^ | 0.507^*^ | -0.164^**^ |
|  | (0.076) | (0.017) | (0.027) | (0.056) | (0.221) | (0.034) |
| Days with birth parents | 0.001 | 0.0003^**^ | 0.0001 | 0.001^*^ | 0.0003^**^ | 0.0001 |
|  | (0.0005) | (0.00004) | (0.0002) | (0.0004) | (0.0001) | (0.0002) |
| Respondent age | -0.030 | 0.019 | -0.005 | -0.027 | 0.013 | -0.008 |
|  | (0.031) | (0.028) | (0.025) | (0.033) | (0.020) | (0.023) |
| Child gender | -0.754 | -0.878 | 0.946^**^ | -0.733 | -0.941 | 0.937^**^ |
|  | (0.711) | (0.624) | (0.282) | (0.730) | (0.548) | (0.279) |
| Sibling(s) in household | 0.649^**^ | 0.355 | -0.103 | 0.672^**^ | 0.272 | -0.135 |
|  | (0.208) | (0.470) | (0.177) | (0.259) | (0.415) | (0.125) |
| Respondent relationship status | -1.570 | -0.932^**^ | 1.720^**^ | -1.560 | -0.656^**^ | 1.620^**^ |
|  | (1.290) | (0.139) | (0.435) | (1.320) | (0.210) | (0.478) |
| Respondent education | 0.126 | -1.110^**^ | 0.377 | 0.137 | -1.060^**^ | 0.350 |
|  | (0.196) | (0.285) | (0.226) | (0.178) | (0.318) | (0.214) |
| Respondent income (2) | -0.175 | 0.086 | -0.257 | -0.192 | 0.244 | -0.269^**^ |
|  | (0.923) | (0.444) | (0.153) | (0.931) | (0.423) | (0.033) |
| Respondent income (3) | -0.018 | 0.377 | -0.631^**^ | -0.020 | 0.578 | -0.694^**^ |
|  | (0.880) | (0.481) | (0.080) | (0.866) | (0.514) | (0.152) |
| Respondent employment | -0.889^**^ | -0.692 | 1.010^**^ | -0.873^**^ | -0.650 | 0.975^**^ |
|  | (0.313) | (0.651) | (0.179) | (0.329) | (0.612) | (0.187) |
| Internalizing *_t_*_-1_ | 0.434^*^ |  |  | 0.435^*^ |  |  |
|  | (0.185) |  |  | (0.188) |  |  |
| Externalizing *_t_*_-1_ |  | 0.444^**^ |  |  | 0.464^**^ |  |
|  |  | (0.136) |  |  | (0.140) |  |
| Prosocial *_t_*_-1_ |  |  | 0.403^**^ |  |  | 0.399^**^ |
|  |  |  | (0.069) |  |  | (0.059) |
| Number of moves x days in care | 0.00005 | -0.001^**^ | 0.0001 |  |  |  |
|  | (0.0002) | (0.0001) | (0.0003) |  |  |  |
| ACE count x days in care |  |  |  | -0.0001 | -0.0005 | 0.0003^**^ |
|  |  |  |  | (0.0002) | (0.0004) | (0.0001) |
| Intercept | 5.380^*^ | 5.100^**^ | 1.970 | 5.070 | 4.750^**^ | 2.660 |
|  | (2.700) | (1.070) | (1.760) | (3.080) | (1.040) | (1.490) |
| Observations | 222 | 222 | 222 | 222 | 222 | 222 |
| Adjusted *R*^2^ | 0.310 | 0.296 | 0.380 | 0.310 | 0.296 | 0.391 |
| *F* statistic | 8.090^**^ | 7.630^**^ | 10.700^**^ | 8.100^**^ | 7.620^**^ | 11.200^**^ |

*Note.* * *p*<.05, ***p*<.01.

Table A5

*Interactions between Days in Care and long-term risk factors. AR-1 (Four-Waves) models used. Standard errors reported in parentheses.*

|  | Number of moves x days in care models | | | ACE count x days in care models | | |
| --- | --- | --- | --- | --- | --- | --- |
|  | Internalizing | Externalizing | Prosocial | Internalizing | Externalizing | Prosocial |
| Number of moves | -0.201 | 0.121 | 0.009 | -0.285^**^ | -0.375 | 0.071 |
|  | (0.228) | (0.218) | (0.121) | (0.112) | (0.196) | (0.057) |
| ACE count | -0.001 | 0.0002 | 0.001^*^ | -0.0004 | 0.0003 | -0.00001 |
|  | (0.0005) | (0.001) | (0.0003) | (0.001) | (0.002) | (0.0001) |
| Days with birth parents | 0.258^**^ | 0.346^**^ | -0.022 | 0.341^*^ | 0.718^**^ | -0.206* |
|  | (0.076) | (0.064) | (0.029) | (0.144) | (0.153) | (0.085) |
| Days in care | 0.001 | -0.0002 | 0.0001 | 0.001 | -0.00001 | 0.0001 |
|  | (0.0004) | (0.0001) | (0.0002) | (0.0004) | (0.0002) | (0.0001) |
| Respondent age | -0.057^*^ | 0.013 | 0.014 | -0.058^**^ | 0.003 | 0.011 |
|  | (0.023) | (0.039) | (0.027) | (0.020) | (0.033) | (0.025) |
| Child gender | -0.871 | -0.676 | 0.963 | -0.891 | ^__^0.818 | 0.946 |
|  | (0.535) | (0.949) | (0.521) | (0.559) | (0.875) | (0.515) |
| Sibling(s) in household | 0.877^**^ | 0.436 | 0.024 | 0.856^**^ | 0.269 | -0.005 |
|  | (0.071) | (0.546) | (0.126) | (0.180) | (0.473) | (0.053) |
| Respondent relationship status | -2.110 | -1.480^**^ | 1.850^**^ | 2.060 | -1.200^**^ | 1.790^**^ |
|  | (1.200) | (0.227) | (0.492) | (1.220) | (0.260) | (0.500) |
| Respondent education | 0.139 | -1.120^*^ | 0.427 | 0.150 | -1.060^*^ | 0.402 |
|  | (0.126) | (0.453) | (0.273) | (0.089) | (0.463) | (0.240) |
| Respondent income (2) | -0.654 | 0.861 | -0.419^*^ | -0.636 | -0.705^**^ | -0.368^**^ |
|  | (1.200) | (0.447) | (0.173) | (1.220) | (0.229) | (0.098) |
| Respondent income (3) | -0.427 | -0.360 | -0.930^**^ | -0.388 | -0.105 | -0.926^**^ |
|  | (1.180) | (0.481) | (0.228) | (1.200) | (0.373) | (0.259) |
| Respondent employment | -1.250^**^ | -0.905 | 0.978^**^ | -1.250^**^ | -0.900 | 0.942^**^ |
|  | (0.167) | (0.714) | (0.246) | (0.216) | (0.646) | (0.232) |
| Internalizing *_t_*_-1_ | 0.449^*^ |  |  | 0.450^*^ |  |  |
|  | (0.185) |  |  | (0.187) |  |  |
| Externalizing *_t_*_-1_ |  | 0.393^**^ |  |  | 0.411^**^ |  |
|  |  | (0.138) |  |  | (0.137) |  |
| Prosocial *_t_*_-1_ |  |  | 0.464^**^ |  |  | 0.461^**^ |
|  |  |  | (0.119) |  |  | (0.104) |
| Number of moves x days in care | -0.0002 | -0.001^**^ | 0.0001 |  |  |  |
|  | (0.0003) | (0.0002) | (0.0003) |  |  |  |
| ACE count x days in care |  |  |  | -0.0002 | -0.001^*^ | 0.0003^*^ |
|  |  |  |  | (0.0003) | (0.0003) | (0.0001) |
| Intercept | 7.770^**^ | 7.130^**^ | 0.596 | 7.710^**^ | 7.120 | 1.120 |
|  | (1.470) | (1.710) | (1.590) | (1.840) | (1.390) | (1.330) |
| Observations | 177 | 177 | 177 | 177 | 177 | 177 |
| Adjusted *R*^2^ | 0.331 | 0.250 | 0.434 | 0.331 | 0.251 | 0.446 |
| *F* statistic | 7.210^**^ | 5.190^**^ | 10.600^**^ | 7.230^**^ | 5.210^**^ | 11.100^**^ |

*Note.* * *p*<.05, ***p*<.01.

**Manuscript interactions for all models**

All marginal effects plots analysed are reported in Tables A6 to A11, including those we do not discuss in the manuscript.

Table A6

*Interactions between number of moves and long-term risk factors (days in care and days with birth parents) on externalizing problems. AR-1 and AR-1 (Four-Waves) models used. Standard errors reported in parentheses.*

|  | Externalizing problems | | | |
| --- | --- | --- | --- | --- |
|  | AR-1 (number of moves x days with birth parents model) | AR-1 (number of moves x days in care model) | AR-1 (Four-Wave) (number of moves x days with birth parents model) | AR-1 (Four-Wave) (number of moves x days in care model) |
| Number of moves | -0.369 | 0.017 | -0.434 | 0.121 |
|  | (0.313) | (0.151) | (0.377) | (0.218) |
| ACE count | 0.0002 | 0.0003** | -0.0003 | -0.0002 |
|  | (0.001) | (0.00004) | (0.002) | (0.0001) |
| Days with birth parents | 0.242** | 0.260** | 0.301** | 0.346** |
|  | (0.010) | (0.017) | (0.063) | (0.064) |
| Days in care | -0.001* | 0.0002 | -0.002 | 0.0002 |
|  | (0.0005) | (0.0002) | (0.001) | (0.001) |
| Respondent age | 0.007 | 0.019 | -0.003 | 0.013 |
|  | (0.024) | (0.028) | (0.019) | (0.039) |
| Child gender | -1.020 | -0.878 | -0.935 | -0.676 |
|  | (0.610) | (0.624) | (0.913) | (0.949) |
| Sibling(s) in household | 0.189 | 0.355 | 0.131 | 0.436 |
|  | (0.518) | (0.470) | (0.548) | (0.546) |
| Respondent relationship status | -0.805** | -0.932** | -1.290** | -1.480** |
|  | (0.100) | (0.139) | (0.271) | (0.227) |
| Respondent education | -1.100** | -1.110** | -1.090 | -1.120* |
|  | (0.307) | (0.285) | (0.585) | (0.453) |
| Respondent income (2) | 0.285 | 0.086 | -0.516* | -0.861 |
|  | (0.343) | (0.444) | (0.221) | (0.447) |
| Respondent income (3) | 0.557 | 0.377 | 0.033 | -0.360 |
|  | (0.482) | (0.481) | (0.484) | (0.481) |
| Respondent employment | -0.726 | -0.692 | -1.010 | -0.905 |
|  | (0.685) | (0.651) | (0.778) | (0.714) |
| Externalizing *_t_*_-1_ | 0.462** | 0.444** | 0.416* | -0.393** |
|  | (0.153) | (0.136) | (0.166) | (0.138) |
| Number of moves x days with birth parents | 0.0001 |  | 0.0002 |  |
|  | (0.0004) |  | (0.001) |  |
| Number of moves x days in care |  | -0.001** |  | -0.001** |
|  |  | (0.0001) |  | (0.0002) |
| Intercept | 6.070** | 5.100** | 8.540** | 7.130** |
|  | (1.010) | (1.070) | (1.450) | (1.710) |
| Observations | 222 | 222 | 177 | 177 |
| Adjusted *R*^2^ | 0.288 | 0.296 | 0.232 | 0.250 |
| *F* statistic | 7.370** | 7.630** | 4.800** | 5.190** |

*Note.* * *p*<.05, ***p*<.01.

Table A7

*Interactions between number of moves and long-term risk factors (days in care and days with birth parents) on internalizing problems. AR-1 and AR-1 (Four-Waves) models used. Standard errors reported in parentheses.*

|  | Internalizing problems | | | |
| --- | --- | --- | --- | --- |
|  | AR-1 (number of moves x days with birth parents model) | AR-1 (number of moves x days in care model) | AR-1 (Four-Wave) (number of moves x days with birth parents model) | AR-1 (Four-Wave) (number of moves x days in care model) |
| Number of moves | -0.298 | -0.197 | -0.310 | -0.201 |
|  | (0.231) | (0.135) | (0.289) | (0.228) |
| ACE count | 0.0004 | 0.001 | 0.001 | 0.001 |
|  | (0.0002) | (0.0005) | (0.0005) | (0.0004) |
| Days with birth parents | 0.173** | 0.163* | 0.252** | 0.258** |
|  | (0.058) | (0.076) | (0.074) | (0.076) |
| Days in care | -0.0004 | -0.0005** | -0.001** | -0.001 |
|  | (0.0004) | (0.0001) | (0.0002) | (0.0005) |
| Respondent age | -0.025 | -0.030 | -0.059** | -0.057* |
|  | (0.033) | (0.031) | (0.011) | (0.023) |
| Child gender | -0.772 | -0.754 | -0.917 | -0.871 |
|  | (0.729) | (0.711) | (0.571) | (0.535) |
| Sibling(s) in household | 0.628** | 0.649** | 0.824** | 0.877** |
|  | (0.222) | (0.208) | (0.104) | (0.071) |
| Respondent relationship status | -1.600 | -1.570 | -2.080 | -2.110 |
|  | (1.260) | (1.290) | (1.210) | (1.200) |
| Respondent education | 0.154 | 0.126 | 0.145 | 0.139 |
|  | (0.162) | (0.196) | (0.097) | (0.126) |
| Respondent income (2) | -0.155 | -0.175 | -0.595 | -0.654 |
|  | (0.901) | (0.923) | (1.180) | (1.200) |
| Respondent income (3) | 0.039 | -0.018 | -0.352 | -0.427 |
|  | (0.823) | (0.880) | (1.080) | (1.180) |
| Respondent employment | -0.892** | -0.889** | -1.270** | -1.250** |
|  | (0.304) | (0.313) | (0.209) | (0.167) |
| Internalizing *_t_*_-1_ | 0.441* | 0.434* | 0.452* | 0.449* |
|  | (0.190) | (0.185) | (0.200) | (0.185) |
| Number of moves x days with birth parents | 0.0002 |  | 0.0001 |  |
|  | (0.0003) |  | (0.0004) |  |
| Number of moves x days in care |  | 0.00005 |  | -0.0002 |
|  |  | (0.0002) |  | (0.0003) |
| Intercept | 5.310 | 5.380* | 8.000** | 7.770** |
|  | (2.850) | (2.700) | (1.570) | (1.470) |
| Observations | 222 | 222 | 177 | 177 |
| Adjusted *R*_2_ | 0.313 | 0.310 | 0.330 | 0.331 |
| *F* statistic | 8.190** | 8.090** | 7.200** | 7.210** |

*Note.* * *p*<.05, ***p*<.01.

Table A8

*Interactions between number of moves and long-term risk factors (days in care and days with birth parents) on prosocial behavior. AR-1 and AR-1 (Four-Waves) models used. Standard errors reported in parentheses.*

|  | Prosocial behavior | | | |
| --- | --- | --- | --- | --- |
|  | AR-1 (number of moves x days with birth parents model) | AR-1 (number of moves x days in care model) | AR-1 (Four-Wave) (number of moves x days with birth parents model) | AR-1 (Four-Wave) (number of moves x days in care model) |
| Number of moves | 0.081 | -0.021 | 0.079 | 0.009 |
|  | (0.056) | (0.166) | (0.088) | (0.121) |
| ACE count | 0.0002 | 0.0001 | 0.0002 | 0.0001 |
|  | (0.0004) | (0.0002) | (0.0003) | (0.0002) |
| Days with birth parents | 0.008 | 0.009 | -0.018 | -0.022 |
|  | (0.024) | (0.027) | (0.023) | (0.029) |
| Days in care | 0.001** | 0.001 | 0.001* | 0.001* |
|  | (0.0001) | (0.001) | (0.0004) | (0.0003) |
| Respondent age | -0.005 | -0.005 | 0.015 | 0.014 |
|  | (0.020) | (0.025) | (0.022) | (0.027) |
| Child gender | 0.978** | 0.946** | 0.992 | 0.963 |
|  | (0.276) | (0.282) | (0.538) | (0.521) |
| Sibling(s) in household | -0.069 | -0.103 | 0.056 | 0.024 |
|  | (0.103) | (0.177) | (0.034) | (0.126) |
| Respondent relationship status | 1.710** | 1.720** | 1.830** | 1.850** |
|  | (0.465) | (0.435) | (0.504) | (0.492) |
| Respondent education | 0.368 | 0.377 | 0.421 | 0.427 |
|  | (0.237) | (0.226) | (0.274) | (0.273) |
| Respondent income (2) | -0.299** | -0.257 | -0.454** | -0.419* |
|  | (0.094) | (0.153) | (0.047) | (0.173) |
| Respondent income (3) | -0.682** | -0.631** | -0.976** | -0.930** |
|  | (0.119) | (0.080) | (0.271) | (0.228) |
| Respondent employment | 1.020** | 1.010** | 0.990** | 0.978** |
|  | (0.156) | (0.179) | (0.204) | (0.246) |
| Prosocial *_t_*_-1_ | 0.407** | 0.403** | 0.466** | 0.464** |
|  | (0.065) | (0.069) | (0.118) | (0.119) |
| Number of moves x days with birth parents | -0.0001 |  | -0.00004 |  |
|  | (0.0001) |  | (0.0001) |  |
| Number of moves x days in care |  | 0.0001 |  | 0.0001 |
|  |  | (0.0003) |  | (0.0003) |
| Intercept | 1.760 | 1.970 | 0.427 | 0.596 |
|  | (1.330) | (1.760) | (1.160) | (1.590) |
| Observations | 222 | 222 | 177 | 177 |
| Adjusted *R*_2_ | 0.380 | 0.380 | 0.433 | 0.434 |
| *F* statistic | 10.700** | 10.700** | 10.600** | 10.600** |

*Note.* * *p*<.05, ***p*<.01.

Table A9

*Interactions between ACE count and long-term risk factors (days in care and days with birth parents) on externalizing problems. AR-1 and AR-1 (Four-Waves) models used. Standard errors reported in parentheses.*

|  | Externalizing problems | | | |
| --- | --- | --- | --- | --- |
|  | AR-1 (ACE count x days with birth parents model) | AR-1 (ACE count moves x days in care model) | AR-1 (Four-Wave) (ACE count x days with birth parents model) | AR-1 (Four-Wave) (ACE count x days in care model) |
| Number of moves | 0.262** | 0.507* | 0.312** | 0.718** |
|  | (0.042) | (0.221) | (0.079) | (0.153) |
| ACE count | 0.001 | 0.0003** | 0.0001 | -0.00001 |
|  | (0.0003) | (0.0001) | (0.0003) | (0.0002) |
| Days with birth parents | -0.328* | -0.329* | -0.353 | -0.375 |
|  | (0.148) | (0.145) | (0.222) | (0.196) |
| Days in care | -0.001** | 0.001 | -0.002* | 0.0003 |
|  | (0.0004) | (0.002) | (0.001) | (0.002) |
| Respondent age | 0.003 | 0.013 | -0.009 | 0.003 |
|  | (0.031) | (0.020) | (0.044) | (0.033) |
| Child gender | -1.030 | -0.941 | -0.938 | -0.818 |
|  | (0.614) | (0.548) | (0.957) | (0.875) |
| Sibling(s) in household | 0.205 | 0.272 | 0.152 | 0.269 |
|  | (0.445) | (0.415) | (0.478) | (0.473) |
| Respondent relationship status | -0.806** | -0.656** | -1.300** | -1.200** |
|  | (0.100) | (0.210) | (0.260) | (0.260) |
| Respondent education | -1.130** | -1.060** | -1.140* | -1.060* |
|  | (0.301) | (0.318) | (0.511) | (0.463) |
| Respondent income (2) | 0.277 | 0.244 | -0.504 | -0.705** |
|  | (0.334) | (0.423) | (0.272) | (0.229) |
| Respondent income (3) | 0.553 | 0.578 | 0.007 | 0.105 |
|  | (0.465) | (0.514) | (0.412) | (0.373) |
| Respondent employment | -0.723 | -0.650 | -1.010 | -0.900 |
|  | (0.685) | (0.612) | (0.751) | (0.646) |
| Externalizing *_t_*_-1_ | 0.457** | 0.464** | 0.408** | 0.411** |
|  | (0.138) | (0.140) | (0.143) | (0.137) |
| ACE count x days with birth parents | -0.00004 |  | -0.00002 |  |
|  | (0.0001) |  | (0.0001) |  |
| ACE count x days in care |  | -0.0005 |  | -0.001* |
|  |  | (0.0004) |  | (0.0003) |
| Intercept | 6.190** | 4.750** | 8.720** | 7.120** |
|  | (1.100) | (1.040) | (1.940) | (1.390) |
| Observations | 222 | 222 | 177 | 177 |
| Adjusted *R*_2_ | 0.287 | 0.296 | 0.231 | 0.251 |
| *F* statistic | 7.370** | 7.620** | 4.780** | 5.210** |

*Note.* * *p*<.05, ***p*<.01.

Table A10

*Interactions between ACE count and long-term risk factors (days in care and days with birth parents) on internalizing problems. AR-1 and AR-1 (Four-Waves) models used. Standard errors reported in parentheses.*

|  | Internalizing problems | | | |
| --- | --- | --- | --- | --- |
|  | AR-1 (ACE count x days with birth parents model) | AR-1 (ACE count moves x days in care model) | AR-1 (Four-Wave) (ACE count x days with birth parents model) | AR-1 (Four-Wave) (ACE count x days in care model) |
| Number of moves | 0.118 | 0.213** | 0.172** | 0.341* |
|  | (0.078) | (0.056) | (0.011) | (0.144) |
| ACE count | 0.001 | 0.001* | 0.0002 | 0.001 |
|  | (0.001) | (0.0004) | (0.001) | (0.0004) |
| Days with birth parents | -0.157 | -0.176** | -0.250* | -0.285* |
|  | (0.087) | (0.066) | (0.116) | (0.112) |
| Days in care | -0.0004 | -0.0001 | -0.001** | -0.0004 |
|  | (0.0004) | (0.001) | (0.0001) | (0.001) |
| Respondent age | -0.023 | -0.027 | -0.051 | -0.058** |
|  | (0.041) | (0.033) | (0.031) | (0.020) |
| Child gender | -0.714 | -0.733 | -0.868 | -0.891 |
|  | (0.792) | (0.730) | (0.604) | (0.559) |
| Sibling(s) in household | 0.654** | 0.672** | 0.812** | 0.856** |
|  | (0.245) | (0.259) | (0.146) | (0.180) |
| Respondent relationship status | -1.570 | -1.560 | -2.090 | -2.060 |
|  | (1.270) | (1.320) | (1.200) | (1.220) |
| Respondent education | 0.153 | 0.137 | 0.197 | 0.150 |
|  | (0.236) | (0.178) | (0.152) | (0.089) |
| Respondent income (2) | -0.191 | -0.192 | -0.626 | -0.636 |
|  | (0.910) | (0.931) | (1.140) | (1.220) |
| Respondent income (3) | -0.069 | -0.020 | -0.436 | -0.388 |
|  | (0.809) | (0.866) | (1.090) | (1.200) |
| Respondent employment | -0.888** | -0.873** | -1.270** | -1.250** |
|  | (0.342) | (0.329) | (0.247) | (0.216) |
| Internalizing *_t_*_-1_ | 0.435* | 0.435* | 0.451* | 0.450* |
|  | (0.181) | (0.188) | (0.177) | (0.187) |
| ACE count x days with birth parents | 0.0001 |  | 0.0001 |  |
|  | (0.0001) |  | (0.0001) |  |
| ACE count x days in care |  | -0.0001 |  | -0.0002 |
|  |  | (0.0002) |  | (0.0003) |
| Intercept | 5.120 | 5.070 | 7.730** | 7.710** |
|  | (3.180) | (3.080) | (1.990) | (1.840) |
| Observations | 222 | 222 | 177 | 177 |
| Adjusted *R*_2_ | 0.311 | 0.310 | 0.334 | 0.331 |
| *F* statistic | 8.130** | 8.100** | 7.290** | 7.230** |

*Note.* * *p*<.05, ***p*<.01.

Table A11

*Interactions between ACE count and long-term risk factors (days in care and days with birth parents) on prosocial behavior. AR-1 and AR-1 (Four-Waves) models used. Standard errors reported in parentheses.*

|  | Prosocial behavior | | | |
| --- | --- | --- | --- | --- |
|  | AR-1 (ACE count x days with birth parents model) | AR-1 (ACE count moves x days in care model) | AR-1 (Four-Wave) (ACE count x days with birth parents model) | AR-1 (Four-Wave) (ACE count x days in care model) |
| Number of moves | -0.003 | -0.164** | -0.037 | -0.206* |
|  | (0.042) | (0.034) | (0.033) | (0.085) |
| ACE count | -0.00003 | 0.0001 | -0.00003 | 0.0001 |
|  | (0.0003) | (0.0002) | (0.0003) | (0.0001) |
| Days with birth parents | 0.040** | 0.044** | 0.064 | 0.071 |
|  | (0.008) | (0.006) | (0.053) | (0.057) |
| Days in care | 0.001** | -0.0003 | 0.001* | -0.00001 |
|  | (0.0001) | (0.0003) | (0.0004) | (0.0001) |
| Respondent age | -0.001 | -0.008 | 0.018 | 0.011 |
|  | (0.026) | (0.023) | (0.030) | (0.025) |
| Child gender | 0.977** | 0.937** | 0.996 | 0.946 |
|  | (0.294) | (0.279) | (0.549) | (0.515) |
| Sibling(s) in household | -0.079 | -0.135 | 0.051 | -0.005 |
|  | (0.119) | (0.125) | (0.039) | (0.053) |
| Respondent relationship status | 1.710** | 1.620** | 1.830** | 1.790** |
|  | (0.473) | (0.478) | (0.501) | (0.500) |
| Respondent education | 0.387 | 0.350 | 0.440 | 0.402 |
|  | (0.226) | (0.214) | (0.266) | (0.240) |
| Respondent income (2) | -0.290** | -0.269** | -0.462** | -0.368** |
|  | (0.089) | (0.033) | (0.061) | (0.098) |
| Respondent income (3) | -0.670** | -0.694** | -0.980** | -0.926** |
|  | (0.121) | (0.152) | (0.283) | (0.259) |
| Respondent employment | 1.020** | 0.975** | 0.988** | 0.942** |
|  | (0.162) | (0.187) | (0.207) | (0.232) |
| Prosocial behavior *_t_*_-1_ | 0.406** | 0.399** | 0.469** | 0.461** |
|  | (0.067) | (0.059) | (0.117) | (0.104) |
| ACE count x days with birth parents | 0.00002 |  | 0.00003 |  |
|  | (0.0001) |  | (0.0001) |  |
| ACE count x days in care |  | 0.0003** |  | 0.0003* |
|  |  | (0.0001) |  | (0.0001) |
| Intercept | 1.720 | 2.660 | 0.326 | 1.120 |
|  | (1.500) | (1.490) | (1.420) | (1.330) |
| Observations | 222 | 222 | 177 | 177 |
| Adjusted *R*_2_ | 0.380 | 0.391 | 0.434 | 0.446 |
| *F* statistic | 10.700** | 11.200** | 10.600** | 11.100** |

*Note.* * *p*<.05, ***p*<.01.
